# Assessing the Comparative Efficacy and Safety of Warfarin and Rivaroxaban for Cancer Associated Thrombosis: Experience From a Resource Limited Setting

**DOI:** 10.1002/cnr2.70105

**Published:** 2025-01-23

**Authors:** Abel Tenaw Tasamma, Tsegab Alemayehu Bukate, Abdulrahim Mehadi, Bereket Tagesse Handiso, Beniam Yohannes Kassa, Eman Omer Hassen, Zekarias Seifu Ayalew, Dawit Habtie Tegegne, Gebeyehu Tessema Azibte, Tigest Abebaw Zewdie, Tinsaye Zergaw Shihur, Yonas Degelo Geremamo, Yeabsira Dessalegne Mesfin, Rediet M. Alemayehu, Amanuel Kassu Asefa, Fozia Abdela, Addisu Melkie

**Affiliations:** ^1^ School of Medicine; College of Health Sciences Addis Ababa University Addis Ababa Ethiopia; ^2^ John H. Stroger Jr. Hospital of Cook County Chicago Illinois USA

**Keywords:** anticoagulants, cancer, thrombosis

## Abstract

**Background:**

Thromboembolic events are a common cause of morbidity and mortality in patients with cancer. While direct‐acting oral anticoagulants (DOACs) have been established as the preferred agents of anticoagulation in most patients with cancer, data in resource‐limited settings is limited.

**Aims:**

The study aims to assess the comparative efficacy and safety of warfarin and rivaroxaban for cancer‐associated thrombosis (CAT) in a resource‐limited setting.

**Methods and results:**

A single‐center retrospective cohort study was conducted on 201 patients who were on follow‐up from September 2021 to August 2023. The patients were categorized into two groups (1) warfarin and (2) rivaroxaban. They were then retrospectively followed for 12 months. The primary endpoint was a composite of recurrence of venous thromboembolism (VTE), major bleeding event, or all‐cause mortality. The Cox regression model was used to compare the outcome of the two groups. The baseline mean (standard deviation) age of the patients was 48.4 (15.0) and 140 (69.7%) of them were female. 41.3% of the participants had one or more comorbidities, and the most common types of cancer were gynecologic (28.9%), hematologic (21.4%), and intra‐abdominal (16.9%). The most common type of thrombosis was deep vein thrombosis (DVT) (77.1%). The primary composite outcome of VTE recurrence, major bleeding event, and all‐cause mortality occurred in 25 (24.3%) patients in the warfarin group and 11 (11.2%) in the rivaroxaban group (hazard ratio (HR), 0.48; 95% confidence interval (CI), 0.24–0.97; *p* = 0.041).

**Conclusion:**

Rivaroxaban was found to be more efficacious and safer than warfarin for patients with CAT in a resource‐limited setting. This finding is congruent with reports from resource‐abundant countries and recommendations from major international societies.

## 1 Introduction

Thromboembolism is a common complication of malignancy reported in as many as 10% of cancer patients [1, 2, 3, 4, 5]. Venous, arterial, microvascular thrombosis, and superficial thrombophlebitis are all associated with malignancy [4]. The mechanism for the occurrence of thrombosis, in addition to traditional risk factors such as hospitalization, surgery, age, and underlying comorbidities, can be due to the cancer itself, treatment of the cancer, or any other cancer‐related risk factors [6, 7, 8].

The thromboembolic events usually occur in the setting of established malignancy. However, they can sometimes be evident before the malignancy is diagnosed [9, 10]. These events are among the leading causes of death in patients with malignancy together with cancer progression and infection [11].

Treatment of cancer‐associated thrombosis (CAT) including the choice of anticoagulant is affected by several factors such as cost and convenience, contraindication for a chosen agent, the presence of renal or liver failure, local availability of drugs, and cancer type and stage. Options for the treatment of CAT include subcutaneous low molecular weight heparin (LMWH), oral factor Xa antagonists, unfractionated heparin (UFH), and vitamin K antagonist (warfarin).

Warfarin, a vitamin K antagonist, has been the most frequently prescribed and preferred oral anticoagulant in middle and low‐income countries, including Ethiopia, for nearly 70 years [12, 13, 14]. However, it has a narrow therapeutic index and significant drug–drug and drug–food interactions which can easily alter its efficacy [13]. In addition, there must be regular international normalized ratio (INR) follow‐ups to maintain its therapeutic range and adjust the dose regularly.

The treatment of CAT in resource‐limited settings is fraught with challenges, largely due to the interplay of various socioeconomic factors. One major obstacle is the high economic burden associated with direct oral anticoagulants (DOACs), which severely limits their availability and consistent use. For instance, a recent study evaluating deep vein thrombosis (DVT) outcomes in Ethiopia found that only 20.6% of the 408 patients studied were receiving rivaroxaban [15].

Warfarin, often used as a more affordable alternative, presents its own set of challenges. Patients frequently exhibit poor anticoagulation control, as reflected in a study from Jimma, which reported a mean time in therapeutic range (TTR) of just 25.03% among patients using warfarin for various indications [16]. Such suboptimal TTR is likely influenced by limited access to healthcare facilities and the prohibitively high cost of regular INR testing, both of which are critical for effective warfarin management.

Several studies have shown that DOACs have superior efficacy for the treatment of CAT [17, 18, 19, 20], and several international guidelines recommend them as the preferred anticoagulants for patients with this condition. However, due to the scarcity of data on the subject, it is not clear whether similar conclusions can be made for our population. Hence, this study tries to answer the question of which anticoagulant is more effective for the management of CAT by comparing the efficacy and safety of the two most commonly utilized anticoagulants in the nation—warfarin and rivaroxaban.

## 2 Methods

### 2.1 Study Design

A single‐center institution‐based retrospective cohort study was done at Tikur Anbessa Specialized Hospital (TASH) between December 2023 and February 2024. All adult patients (age ≥ 18 years of age) with histologically confirmed solid and hematologic malignancies who developed newly diagnosed CAT and were on follow‐up at TASH from September 2021 to August 2023 were included in the study.

Patients with one or more of the following were excluded from the study: (1) patients who did not take any anticoagulation, (2) patients who took anticoagulants other than warfarin or rivaroxaban, (3) patients with less than 1 week of follow‐up, (4) patients with prior history of venous thromboembolism (VTE), (5) patients who were already taking anticoagulants for indications other than CAT, (6) patients who started taking anticoagulants more than 30 days after the date of diagnosis of CAT, and (7) patients with incomplete data. Venous thromboembolism was defined as any venous thrombosis unequivocally diagnosed with an appropriate imaging study and included both usual and unusual site thromboses. Discontinuation of medication was defined as the presence of a gap of > 7 days in therapy.

### 2.2 Study Procedure

Eligible patients were categorized into one of two groups: (1) warfarin or (2) rivaroxaban based on the initial anticoagulant prescribed for them. They were then followed until one of the following occurs: (1) primary composite outcome, (2) anticoagulation discontinuation, (3) crossover to another anticoagulant, (4) loss to follow‐up, (5) 12 months since the start of anticoagulation, or (6) end of the study period (August 2023). A structured data abstraction tool was used for data collection, and data was obtained through chart review (both electronic medical records and medical charts) and telephone interviews.

### 2.3 Primary Endpoint

The primary endpoint used was a composite of recurrence of VTE, major bleeding events, or all‐cause mortality. For major bleeding events, the International Society on Thrombosis and Hemostasis (ISTH) definition was used [21].

### 2.4 Statistical Analysis

We included all eligible patients from the study site, and therefore, sample size calculation was not performed. Baseline characteristics of patients were summarized using descriptive analysis such as frequencies, means, medians, percentages, standard deviations, and interquartile ranges (IQR). To find out significant differences between the two groups at baseline, the chi‐square test was used for categorical variables, and independent samples Student's *t*‐test was used for normally distributed continuous variables, and the Mann–Whitney *U* test was used for non‐normally distributed continuous variables. The survival experience of the two groups was compared using the Cox regression model and the Kaplan–Meier survival function using the log‐rank test. All analyses were done using Statistical Package for the Social Sciences (SPSS) version 26.

## 3 Results

**FIGURE 1:**
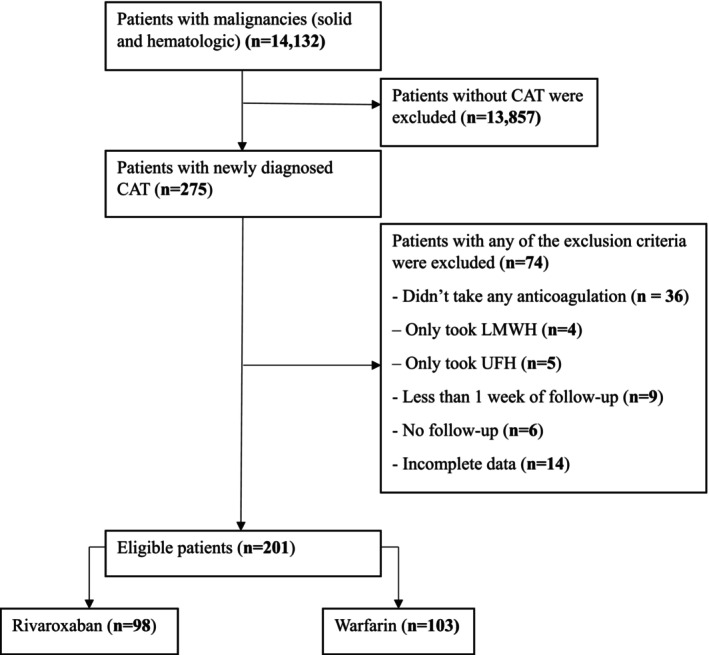
Patient selection flowchart.

Out of a total of 14 132 patients with histologically confirmed cancer, only 275 patients developed venous thrombosis during follow‐up, making the prevalence of thrombosis in patients with cancer 1.95%. After excluding patients who did not fulfill the exclusion criteria, a total of 201 patients (98 in the rivaroxaban and 103 in the warfarin group) were included in the final analysis (Figure 1).

### 3.1 Baseline Characteristics

The baseline mean (SD) age was 48.4 (15.0) and 140 (69.7%) of the patients were female. 83 (41.3%) of the participants had one or more comorbidities, the most common ones being hypertension, HIV, and diabetes mellitus, accounting for 37.4%, 22.9%, and 19.3% of cases, respectively. The 3 most common types of cancer were gynecologic (28.9%), hematologic (21.4%), and intra‐abdominal (16.9%) and the median (IQR) Khorana score was 1 (0–2). The most frequently received modality of treatment for cancer was chemotherapy with 76 (37.8%) patients having taken it. The most common type of thrombosis was DVT (77.1%) and the median (IQR) time for the development of thrombosis was 3.30 (0.11–14.20) months. There was a significant baseline difference between the two groups in terms of the CCI, proportion of patients with hematologic malignancies, type of treatment for cancer, time‐to‐thrombosis, and type of thrombosis (Table 1).

**TABLE 1:** Baseline characteristics of patients.

Characteristics	Warfarin (*n* = 103)	Rivaroxaban (*n* = 98)	Group differences (*p*)	Overall (*n* = 201)
Age (years)—mean (SD)	46.4 (14.2)	50.5 (15.6)	0.053	48.4 (15.0)
Female sex—*n* (%)	69 (67.0)	71 (72.4)	0.492	140 (69.7)
Overall comorbidities—*n* (%)	45 (43.7)	38 (38.8)	0.573	83 (41.3)
Hypertension—*n* (%)	15 (33.3)	16 (42.1)	0.552	31 (37.4)
HIV—*n* (%)	11 (24.4)	8 (21.1)	0.917	19 (22.9)
Diabetes mellitus—*n* (%)	12 (26.7)	4 (10.5)	0.115	16 (19.3)
CCI—median (IQR)	5 (2–6)	6 (3–7)	0.025	6 (3–7)
*Type of cancer*				
Gynecologic—*n* (%)	23 (22.3)	35 (35.7)	0.053	58 (28.9)
Hematologic—*n* (%)	29 (28.2)	14 (14.3)	0.026	43 (21.4)
Intra‐abdominal—*n* (%)^a^	19 (18.4)	15 (15.3)	0.685	34 (16.9)
Khorana score—median (IQR)	1 (0–2)	1 (0–2)	0.704	1 (0–2)
*Type of treatment for cancer*				
Chemotherapy	47 (45.6)	29 (29.6)	0.015	76 (37.8)
Palliative care alone	13 (12.6)	8 (8.2)		21 (10.4)
Other treatment strategies	43 (41.7)	61 (62.2)		104 (51.7)
Time to thrombosis (months)—median (IQR)	2.00 (0–12.00)	5.12 (1.01–18.00)	0.002	3.30 (0.11–14.20)
*Type of thrombosis*				
DVT	74 (71.8)	81 (82.7)	0.010	155 (77.1)
PTE	13 (12.6)	14 (14.3)		27 (13.4)
Others	16 (15.5)	3 (3.1)		19 (9.5)

Abbreviations: CCI, Charlson Comorbidity Index; DVT, deep vein thrombosis; HIV, human immunodeficiency virus; IQR, interquartile range; PTE, pulmonary thromboembolism; SD, standard deviation.

^a^ Excluding intraabdominal lymphomas.

### 3.2 Primary Outcome

**FIGURE 2:**
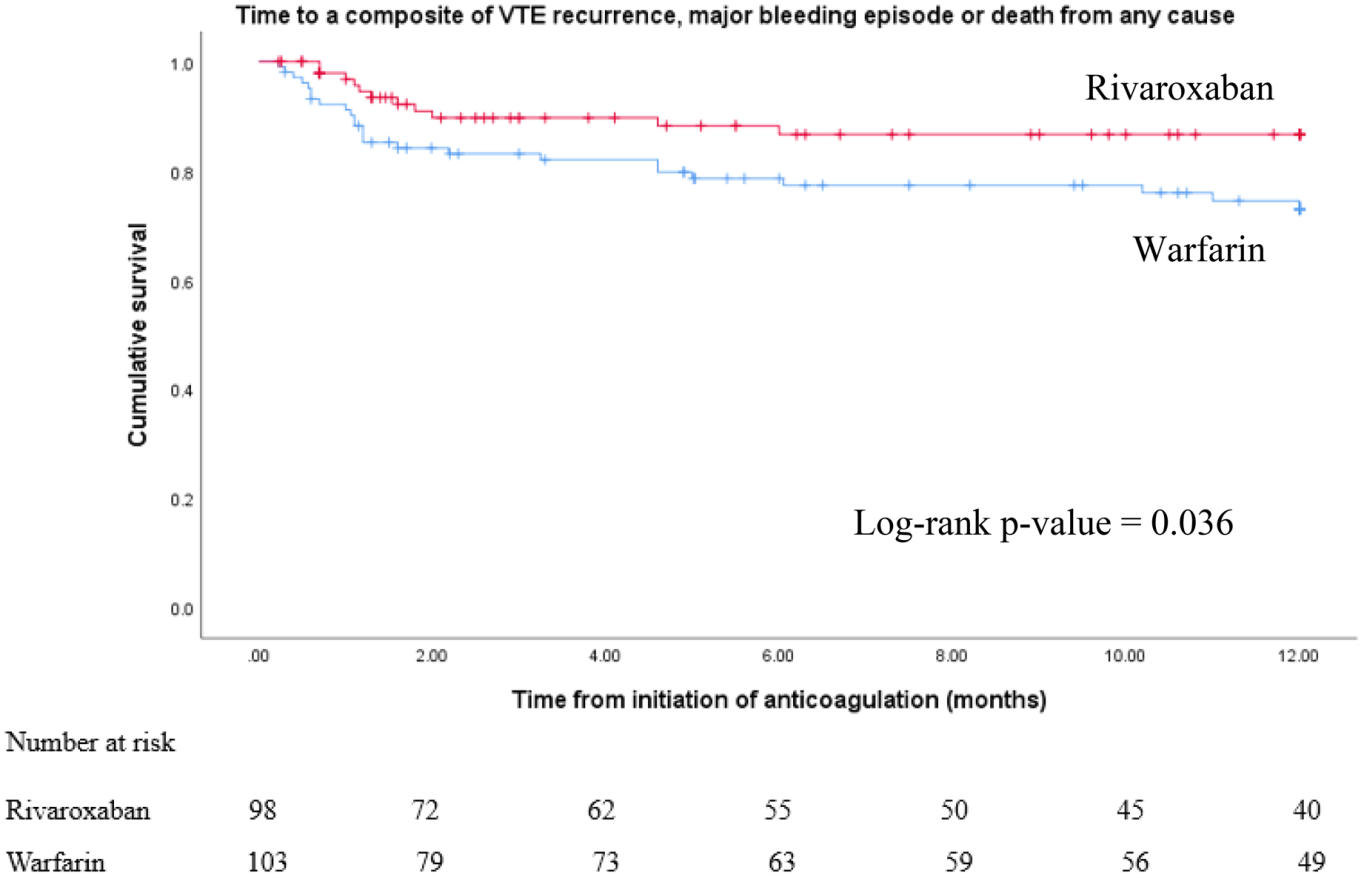
Kaplan–Meier curve for the primary composite outcome.

The primary composite outcome of VTE recurrence, major bleeding event, and all‐cause mortality occurred in 25 (24.3%) patients in the warfarin group and 11 (11.2%) in the rivaroxaban group (adjusted hazard ratio (AHR), 0.48; 95% confidence interval (CI), 0.24–0.97; *p* = 0.041). The Cox regression model was used to adjust for factors that showed significant (*p* < 0.05) group differences at baseline between the two groups. These factors were (1) Charlson Comorbidity Index (CCI), (2) type of cancer, (3) type of treatment for cancer, (4) time to thrombosis and (5) type of thrombosis. However, upon analysis of the individual outcome measures, only all‐cause mortality showed statistically significant differences between the two groups (Table 2 and Figure 2).

**TABLE 2:** Primary outcome.

Outcome	Warfarin *n* (%)	Rivaroxaban *n* (%)	AHR (95% CI)	*p*
Composite of VTE recurrence, major bleeding episode, or death from any cause	25 (24.3)	11 (11.2)	0.48 (0.24–0.97)	0.041
VTE recurrence	6 (5.8)	2 (2.0)	0.38 (0.08–1.86)	0.230
Major bleeding episode	5 (4.9)	2 (2.0)	0.43 (0.08–2.21)	0.310
Death from any cause	19 (18.4)	7 (7.1)	0.41 (0.17–0.97)	0.043

The final outcome of the patients was as follows: 36 patients developed the composite endpoint; 89 patients were followed for a full 1 year but did not develop the endpoints of interest; 23 patients discontinued their anticoagulation; 14 patients were lost to follow‐up; 15 patients crossed over (11 from warfarin to rivaroxaban and 4 from rivaroxaban to warfarin) and the follow‐up of 24 patients was terminated on August 31 (before reaching 1 year of follow‐up).

### 3.3 Subgroup Analysis

A subgroup analysis of the primary endpoint has been done for sex and age.

Age

Less than 50—AHR = 0.38 (95% CI, 1.00–0.54), *p* = 0.54.

50 or more—AHR = 0.735 (95% 0.27–1.97), *p* = 0.54.

Gender

Male—AHR = 0.90 (95% CI 0.29–2.77), *p* = 0.858.

Female—AHR = 0.347 (95% CI 0.14–0.88), *p* = 0.026.

## 4 Discussion

This study sought to compare the effectiveness of rivaroxaban and warfarin in patients diagnosed with CAT in Ethiopia. The study is unique in that it was done in a resource‐limited setting where the optimal anticoagulation strategy remains largely unexplored. Our findings revealed that the composite outcome of VTE recurrence, major bleeding events, and death from any cause was significantly lower in the rivaroxaban group. This highlights a potential overall benefit of rivaroxaban in managing CAT in resource‐limited settings.

Our study utilized a composite endpoint comprising VTE recurrence, major bleeding events, and all‐cause mortality. When analyzed individually, only the difference in all‐cause mortality reached statistical significance. However, it is worth noting that there was a trend toward better outcomes for both VTE recurrence and major bleeding events in the rivaroxaban group. This suggests that future studies with larger sample sizes may reveal statistically significant differences between the groups. As most of the studies, we referenced evaluated individual endpoints rather than composite ones, this difference in approach should be taken into account when interpreting comparisons.

Certain differences in baseline characteristics between the two groups may have contributed to the poorer outcomes observed in the warfarin group. For example, patients in the warfarin group were more likely to (i) have hematologic malignancies, (ii) be receiving chemotherapy, and (iii) be on palliative care without cancer‐directed treatment. We addressed this potential bias by adjusting the HR using these variables as covariates in the Cox regression model.

For nearly two decades, LMWH has been the preferred treatment over warfarin for CAT. This was driven by pivotal studies, including the landmark CLOT study, which demonstrated LMWH's superiority in reducing VTE recurrence without increasing the risk of bleeding [22]. Guidelines from esteemed organizations like the American Society of Clinical Oncology (ASCO) and ISTH have recommended LMWH as the first‐line treatment for CAT [23, 24].

In recent years, several large‐scale, high‐quality international studies have underlined the safety and efficacy of DOACs, including rivaroxaban, in the management of CAT. For instance, one pivotal study, the HOKUSAI VTE‐Cancer trial, involving 1050 patients with active cancer and VTE, compared edoxaban with delteparin. The results of the study indicated that oral edoxaban was noninferior to delteparin on composite outcomes of recurrent VTE and major bleeding [25]. The findings were echoed in subsequent systematic reviews and meta‐analyses. One such study which included 6 randomized control trials (RCTs) enrolling 3690 patients with CAT, revealed that compared to LMWHs, DOACs significantly decreased the risk of CAT recurrence, with non‐significant increases in the risk of major bleeding, a significant increase in the risk of clinically relevant nonmajor bleeding and no difference in all‐cause mortality rates [26]. These findings are reflected in guidelines, with organizations such as ASCO, updating their recommendations to endorse DOACs as first‐choice anticoagulants for VTE treatment in patients with cancer, albeit with caveats regarding their use in patients with high bleeding risks or gastrointestinal cancers [26, 27].

Direct comparisons assessing the effectiveness and safety of vitamin K antagonists and DOACs in cancer patients are relatively limited. However, one meta‐analysis, comparing DOAC versus vitamin K antagonists in patients with cancer and atrial fibrillation, revealed lower rates of stroke or systemic embolism, ischemic stroke, VTE, and myocardial infarction (MI). Additionally, DOAC users also exhibited lower rates of major and intracranial bleedings [28]. A separate cohort study that assessed the comparative effectiveness of DOACs, LMWH, and warfarin in patients with CAT revealed a lower risk of VTE recurrence, major bleeding, and mortality in patients with DOACs [17]. This suggests that DOACs may offer a more favorable balance of efficacy and safety in cancer patients compared to vitamin K antagonists.

Our findings align with several international studies that have underlined the safety and efficacy of DOACs, including rivaroxaban, in the management of CAT. Conducted in resource‐limited settings, our study faced challenges, including limitations to the availability of vast patient data. While the effect sizes for individual outcomes might be clinically meaningful, they did not reach statistical significance due to constraints of our sample size. However, the effect is reflected in the composite outcome, which by pooling various events, revealed the broader clinical impact that achieved statistical significance. Our study contributes a valuable insight into the limited body of evidence from resource‐constrained environment, by offering a perspective from Ethiopia. It indicates that rivaroxaban might offer a viable and safer option compared to warfarin, aligning with global trends in anticoagulation therapy. The findings have clinical implications which can affect patient care and clinical practice, particularly in resource‐limited settings. The data suggests that rivaroxaban has the potential to alleviate the burden on healthcare systems in resource‐limited settings by reducing the need for intensive monitoring and management of complications associated with warfarin therapy. The potential benefit, however, is contingent on drug availability and cost of the drug. Nevertheless, the study's findings should prompt greater utilization of DOACs, given the apparent low usage reflected by the nearly equal number of patients taking both drugs at baseline.

Another notable finding from the study is warfarin being used more frequently than rivaroxaban for CAT associated with hematologic malignancies. The possible explanation for this the fact that patients with hematologic malignancies appear sicker than those with solid malignancies and they are frequently hospitalized making physicians wary of the bleeding risk associated with DOACs.

A principal strength of this study lies in its focus on a resource‐limited setting, providing valuable insights into the management of CAT where evidence is scarce. However, our investigation encountered several limitations. First, a higher dropout rate was observed during follow‐up underscoring the necessity for a more substantial study population and an extended follow‐up period to robustly ascertain outcomes. In addition, in our study, there were significant variations in patient characteristics between groups, including the type of malignancy, treatment modalities, and the nature and timing of thrombotic events. These disparities could affect the study's conclusion and further highlight the need for employing uniform criteria for participant selection in future research.

Future studies should prioritize prospective designs and RCTs to compare the effectiveness and safety of different available DOACs and LMWH in resource‐limited settings. This will provide clearer insight into the optimal anticoagulation strategy. Furthermore, investigations into the cost‐effectiveness as well as patient‐centered outcomes like quality of life and adherence to therapy are essential to inform comprehensive care plans for patients with CAT.

In summary, our research offers preliminary insight supporting the preference for rivaroxaban over warfarin for the management of CAT in Ethiopia. The findings carry significant implications for clinical practice in similar settings, potentially guiding a shift towards more effective and safer anticoagulation strategies. It's imperative that ongoing research efforts are intensified to comprehensively delineate the optimal management strategy of CAT in resource‐limited environments globally, ensuring that therapeutic approaches are both scientifically sound and practically feasible.

## Author Contributions

Conceptualization: A.T.T., B.Y.K., T.A.B. and R.M.A.; Methodology: A.T.T., A.K.A., Z.S.A., T.A.Z. and T.Z.S.; Investigation: B.T.H., E.O.H. and D.H.T.; Formal analysis: A.T.T., Y.D.G., G.T.A. and Y.D.M.; Writing – original draft, A.T.T., A.M. and B.Y.K.; Writing – review and editing, F.A. and A.M.; Supervision: F.A. and A.M.

## Ethics Statement

Ethical approval was obtained from the Institutional Review Board (IRB) of Addis Ababa University (AAU), Department of Internal Medicine. Written or verbal consent was not required as data was collected from patients' medical records and not directly from patients.

## Conflicts of Interest

The authors declare no conflicts of interest.

## Data Availability

The data that support the findings of this study are available on request from the corresponding author.
